# Meta-work: *how* we research is as important as *what* we research

**DOI:** 10.3399/bjgp22X718757

**Published:** 2022-02-25

**Authors:** Yvette Pyne, Stuart Stewart

**Affiliations:** Centre for Academic Primary Care, Bristol Medical School, University of Bristol, Bristol.; Centre for Primary Care and Health Services Research, Division of Population Health, Health Services Research & Primary Care, University of Manchester, Manchester; Research Ambassador, Rochdale Care Organisation, Northern Care Alliance.

## CROSSING THE ‘DOI FINISH LINE’

*‘Manuscripts, like sea turtle hatchlings, face many hazards during their harrowing journey from the nest to the open sea, and many never make it.’*^1^ With MEDLINE indexing an average of two new citations every minute,^2^ the volume of successful medical research publication belies the extent of academic work that never reaches the DOI finish line.^3^ Almost half of all published abstracts do not lead to published results and papers,^4^ and there are likely many reasons why this happens: whether the research itself was not finished because of funding problems, the research was completed but not written up, or the researchers could not find a journal that would publish their findings. A common thread weaving through these issues relates to the availability of time and the ability to use that time to produce only high-quality writing. Often the brightest minds of academia find that they are instead doing low-value work such as form-filling, reading and responding to faculty email chains, and ‘copy-pasting’ ideas between multiple similar documents; this bureaucracy has become such a recognised issue that it is now the subject of a national governmental review.^5^ Even when researchers engage in typically higher-value work such as reading and interpreting academic literature, the process can be overly iterative with too much reliance on human memory of the content of hundreds of papers rather than using external digital tools to capture meaningful notes; notes that can later be found and quickly re-used to create new, original writing without risk of plagiarism.

## THE DARK ART OF SUCCESSFUL PUBLICATION

This article aims to provide key pointers on how we, as early-career academics, have addressed this issue through the selective use of digital tools that help us streamline our research workflow — from reading and assimilating existing literature to capturing new insights and generating new ideas. Through adopting these ourselves in the last year, we have produced several first-author publications, are working on two books, and are chief investigators on three different projects despite also spending half of our week in clinical GP training.

Formal clinical academic teaching, when it is offered, often focuses on specific methodologies such as qualitative or quantitative analyses and the use of statistical software packages with less focus on the larger picture of to how make the most of our academic time. Early-career researchers looking for academic inspiration are invited to *‘Just find a research question’*. This apparently simple suggestion can be challenging to fulfil for those with less experience and research training, and instead we may find ourselves working on projects related to our supervisor’s interests. Even if we can formulate our own research questions, the process of turning them into high-quality research and ultimately publications can appear an impenetrably dark art.

## PERSONAL KNOWLEDGE GRAPHS

Niklas Luhmann, an important and prolific 20th-century social theorist, had no issues producing and sharing high-quality ideas: he wrote and published over 400 articles and 70 books, which included core texts across several disciplines during his late-starting academic career. The quality, volume, and breadth of his output has been credited to his ‘ *Zettelkasten*’ (German for ‘slip box’) note-taking system.^6^ This slip box was filled with thousands of index cards that were linked together with a metadata-based indexing system and, through it, he created a virtual academic conversation partner and confidant long before the internet or personal computers were even conceived of. This tool enabled him to not only access his extensive notes but, by journeying through them, he was also able to formulate new and innovate insights across multiple disciplines and beyond traditional academic boundaries.

Today, we are familiar with interlinked pockets of information in the form of hyperlinks on webpages such as Wikipedia. However, in the context of personal knowledge systems, the last year has seen an explosion of ‘Personal Knowledge Graph (PKG)’ tools such as ‘Roam Research’, ‘Obsidian’, and ‘Notion’, which digitise and personalise this powerful concept. Using bidirectional links, these tools connect notes in ways that sit between traditional word processors and more advanced databases with graphical functions. PKGs represent an evolution in knowledge aggregation and assimilation through the ability to ‘visualise’ personal learning in graphical form. Rather than our hard-won notes being lost in personal silos of projects, folders, and just the passing of time, such graphs allow researchers to literally pan out and visualise their knowledge as a network, revealing nascent and serendipitous connections between percolating notes of ideas, insights, concepts, quotes, and questions.

**Figure 1.**
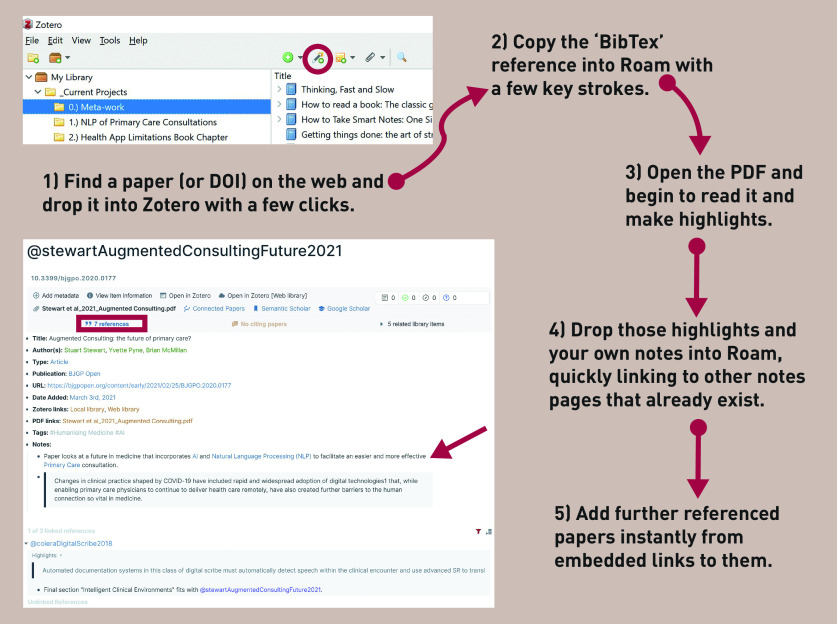
*Academic workflow combining Roam Research (a PKG) with Zotero (a reference manager) and zoteroRoam (an open-source user-built JavaScript extension).*

Figure 1 shows the frictionless workflow combining Roam Research (a PKG) with Zotero (a reference manager) and zoteroRoam (an open-source user-built JavaScript extension). These tools seamlessly link an original paper, all of its relevant metadata, the papers it references, and a researcher’s personal and hyperlinked notes. The researcher can refer to any paper or concept anywhere in their personal knowledge graph and it will automatically link back to it under ‘linked references’.

These tools are ultimately evolving to facilitate fully collaborative academic working where groups of researchers can develop ideas in real time and asynchronously, free of geographical constraints, faculty silos, and the technical limitations associated with traditional word-processor document sharing. While PKGs themselves are too new to have a body of evidence associated with them, when asked about the importance of personal knowledge management (PKM), academics described PKM as improving individual productivity and helping identify knowledge gaps.^7^ However, the same study highlighted that the academics at the time lacked access to this new generation of PKG tools to frictionlessly access and enhance their own ‘personal knowledge databases’. We postulate that, as with artificial intelligence and clinical medicine, these new knowledge tools will not replace human academics, but academics who use them effectively can storm ahead of those who don’t, both in the quality and quantity of their output.

## A READING LIST FOR SMARTER ACADEMIC WORKING

Along with using PKGs, there are many valuable resources available that have helped us to refine how we work (meta-work). They cover skills as simple as ‘how to read a book’^8^ or, while reading that book or another literature source, ‘how to take smart notes’^9^ from them. Once ready to begin writing, there are good books that have taught us how to write a lot^1^ and to write well.^10^^,^^11^ Finally, within the wider context of ‘achieving flow’ and making the most of our limited time, there are books and courses on the art of ‘getting things done’^12^ and on empowering us to do our best ‘deep work’.^13^

Early-career researchers are ‘pluripotent’ with endless opportunities to learn and grow from. By dedicating some thought to how we research as well as what we research, we can make the most of our finite time and energy, and have even more fulfilled and productive academic careers.
